# Senescence Induction by Combined Ionizing Radiation and DNA Damage Response Inhibitors in Head and Neck Squamous Cell Carcinoma Cells

**DOI:** 10.3390/cells9092012

**Published:** 2020-09-01

**Authors:** Clara Dobler, Tina Jost, Markus Hecht, Rainer Fietkau, Luitpold Distel

**Affiliations:** Department of Radiation Oncology, Universitätsklinikum Erlangen, Friedrich-Alexander-Universität Erlangen-Nürnberg, D-91054 Erlangen, Germany; Clara_Dobler@web.de (C.D.); tina.jost@uk-erlangen.de (T.J.); markus.hecht@uk-erlangen.de (M.H.); rainer.fietkau@uk-erlangen.de (R.F.)

**Keywords:** HNSCC, DNA damage response inhibitor, kinase inhibitor, senescence, ATM, ATR, DNAPK, ionizing radiation, radiosensitivity, homologous recombination

## Abstract

DNA damage response inhibitors (DDRi) may selectively enhance the inactivation of tumor cells in combination with ionizing radiation (IR). The induction of senescence may be the key mechanism of tumor cell inactivation in this combinatorial treatment. In the current study the effect of combined IR with DDRi on the induction of senescence was studied in head and neck squamous cell carcinoma (HNSCC) cells with different human papilloma virus (HPV) status. The integrity of homologous recombination (HR) was assessed in two HPV positive, two HPV negative HNSCC, and two healthy fibroblast cell cultures. Cells were treated with the DDRi CC-115 (DNA-dependent protein kinase, DNA-pK; dual mammalian target of rapamycin, mTor), VE-822 (ATR; ataxia telangiectasia and Rad3-related kinase), and AZD0156 (ATM; ataxia telangiectasia mutated kinase) combined with IR. Effects on senescence, apoptosis, necrosis, and cell cycle were analyzed by flow cytometry. The fibroblast cell lines generally tolerated IR or combined treatment better than the tumor cell lines. The ATM and ATR inhibitors were effectively inducing senescence when combined with IR. The DNA-PK inhibitor was not an important inductor of senescence. HPV status and HR activity had a limited influence on the efficacy of DDRi. Induction of senescence and necrosis varied individually among the cell lines due to molecular heterogeneity and the involvement of DNA damage response pathways in senescence induction.

## 1. Introduction

Kinase inhibitors play an increasingly important role in cancer treatment [1]. The combination of DNA damage response inhibitors (DDRi) with ionizing radiation (IR) is of particular interest, as the induction of DNA damage is the most important biological effect of IR [2]. The repair of DNA double-strand breaks (DSB) is of certain interest, as homologous recombination (HR) is often impaired in tumors [3]. Thus, DNA damage in tumor can often only be repaired by non-homologous end joining (NHEJ), whereas the surrounding healthy tissue can use both HR and NHEJ. It is proposed that inhibitors of the NHEJ will specifically target tumor cells. While normal tissue cells are capable of repairing DNA damage by HR, radiation-induced DSB cannot be repaired in tumor cells due to drug-induced NHEJ inhibition. Thus, the combination of DDRi with IR seems to be a promising strategy in cancer treatment [1,2,3]. Head and neck squamous cell carcinoma (HNSCC) may be a suitable tumor entity, as clinical treatment typically contains radiation therapy and these tumors still have a poor prognosis despite the progress made in recent years [4]. The most important predictor of the success of radiation therapy treatment in HNSCC is human papilloma virus (HPV) status. HPV positive tumors respond significantly better to radiochemotherapy than HPV negative tumors [5,6,7]. In radiation therapy it is important to consider both the effect on the tumor and the surrounding normal tissue. As the induction of cell death must especially appear in the tumor and not in the surrounding healthy tissue, both tumor and healthy tissue cells have to be studied. In most published analyses, cell death is studied by colony forming assays, apoptosis, or necrosis. However, the induction of senescence also has an important influence on the success of tumor treatment. Although metabolically active, senescent cells are cell cycle arrested and could help to reduce initial tumor growth or even lead to immune response-mediated tumor regression [8]. In normal tissue, senescence leads to increased fibrosis and treatment related late toxicity.

In the following study, the effect of the kinase inhibitors AZD0156, inhibitor of Ataxia telangiectasia mutated kinase (ATM), VE-822, inhibitor of Ataxia telangiectasia and Rad3-related kinase (ATR) and CC-115, inhibitor of both dual mammalian target of rapamycin (mTor) and DNA-dependent protein kinase (DNA-PK), was assessed. The effect of the inhibitors in combination with IR on HPV positive and negative HNSCC tumor cells was compared to healthy tissue fibroblasts. The study focuses on cell inactivation by senescence and possible interaction effects of combined IR and DDRi.

## 2. Materials and Methods

### 2.1. Cell Culture

Cal33, HSC4, UM-SCC-47, and UD-SCC-2 are HNSCC cells and were kindly provided by Dr. Thorsten Rieckmann (University Medical Center Hamburg-Eppendorf, Germany). As described before [5,9,10], Cal33 and HSC4 are HPV-negative, whereas UM-SCC-47 and UD-SCC-2 contain sequences of the most common high-risk type HPV-16. Human skin fibroblasts SBLF7 and SBLF9 were derived from healthy caucasian males and cultured subsequently. HNSCC were cultured in Dulbecco’s MEM (DMEM; Gibco, Waltham, MA, USA) supplemented with 10% FBS (Biochrom, Berlin, Germany) and 5% penicillin-streptomycin (Gibco, Waltham, MA, USA). Fibroblasts were cultured in F-12 (Gibco, Waltham, MA, USA) supplemented with 15% FBS (Biochrom, Berlin, Germany), 5% penicillin-streptomycin (Gibco, Waltham, MA, USA), and 5% NEA (Biochrom, Berlin, Germany). All cell cultures were grown at 37 °C and 5% CO_2_ in a humidified atmosphere.

### 2.2. Homologous Recombination by Immunofluorescence Microscopy

Cells were seeded on cover slides and cultured to 90% confluence maximum. Culture medium was exchanged, and half of the samples were treated with 5 µmol/L of CC-115 DNA-PK and mTor inhibitor. After 24 h of incubation at 37 °C, cells were irradiated with 10 Gy by an ISOVOLT Titan X-ray generator (GE, Ahrensburg, Germany). After another 4 h, slides were fixed and permeabilized with 4% formaldehyde and 0.1% Triton X-100/PBS for 15 min. Slides were then blocked with 1% BSA overnight. Staining with primary antibodies mouse anti-γH2AX (1:1500, Merck, Darmstadt, Germany) and rabbit anti-Rad51 (1:250, abcam, Cambridge, UK) was carried out overnight at 8 °C [11]. Slides were further stained with secondary antibodies AlexaFluor488 goat anti-mouse and AlexaFluor594 chicken anti-rabbit (Invitrogen, Eugene, OR, USA). DAPI was applied for DNA staining (10236276001, Sigma Aldrich, St. Louis, MO, USA). Cover slides were mounted onto glass slides using Vectashield (Vector Laboratories, Burlingame, CA, US) and images were acquired by a Zeiss Imager Z2 fluorescence microscope (Zeiss, Oberkochen, Germany). Foci were quantified using Biomas Software (Version V3.07/2012, MSAB, Erlangen, Germany).

### 2.3. Combined Assay for Senescence, Cell Cycle, Apoptosis, and Necrosis by Flow Cytometry

Cells were seeded in T25 flasks (day 0). Numbers of sewed cells were adapted to the proliferation rates of the cell lines (Supplementary Figure S1). After 24 h, the medium was changed to a serum-reduced culture medium (2% FBS). Cells were then treated with AZD0156 ATM kinase inhibitor (Selleckchem, Houston, TX, USA; S8375), CC-115 DNA-PK and mTOR inhibitor (Selleckchem, Houston, TX, USA; S7891), or VE-822 ATR inhibitor (Selleckchem, Houston, TX, USA; S7102). For AZD0156 and CC-115, concentrations used were 0, 0.1, and 0.25 µmol/L. VE-822 was used at concentrations of 0, 0.02, and 0.05 µmol/L. 

Half of the cell culture flasks were irradiated by 2 Gy ionizing radiation (IR) 3 h after adding the drug on day 1. Change of cell culture medium and treatment with the respective inhibitors and concentrations was repeated on day 4. Cells were analyzed by flow cytometry on day 10. 

Consequently, cells including the supernatant were harvested. After centrifugation (8 min, 20 °C, 180 g) and removing of the supernatant, cells were resuspended in 400 µL of cell culture medium (2% FBS), treated with 4 µL of Bafilomycin A1 (BAF 1A), *Streptomyces griceus* (Millipore, Burlington, MA, USA), and incubated at 37 °C and 5% CO_2_. Bafilomycin A1 in this assay is used to inhibit the activity of endogenous beta-galactosidase by neutralizing the acidic pH of the lysosomes [12]. This allows to distinguish hydrolyzation of C_12_FDG by senescent-associated beta-galactosidase (SA-β-Gal) showing activity at the provided unideal pH [13]. Cells were incubated for 30 min in Bafilomycin A1, then 2 µL of Hoechst 33342 (Molecular Probes, Eugene, OR, USA) was added and incubation resumed for another 30 min before adding 0.5 µL C_12_FDG (Invitrogen, Carlsbad, CA, USA). Incubation was continued for another hour and then samples were centrifuged (6 min, 20 °C, 400 g), the supernatant was removed and the pellet resuspended with 200 µL of ice cold Ringer’s solution (Fresenius Kabi, Bad Homburg, Germany). Then, 10 µL of a 1:1 mixture of APC Annexin and 7AAD (BD Biosciences, Franklin Lakes, NJ, USA) was added and the cells incubated light-protected on ice for 30 min. Afterwards, cells were centrifuged again (6 min, 20 °C, 400g), resuspended in Ringer’s solution after removing the supernatant and analyzed by a CytoFLEX S flow cytometer (Beckmann Coulter, Brea, CA, USA) using PB450, FITC, PerCP, and APC channels. Data evaluation was performed using Kaluza Analysis software (Version 4.1 07/2018, Beckman Coulter, Brea, CA, USA).

### 2.4. Gating Strategy for Flow Cytometry

Cells were identified by the forward and sideward scatter and doublets were excluded by Hoechst staining and its area to height ratio. Apoptotic cells and necrotic cells were assigned by Annexin APC and 7AAD staining. Senescent cells were identified by BAF 1A and C_12_FDG treated cells. Senescent cells were detected among live cells only. The gate for C_12_FDG fluorescence was set up based on the appearance of a second C_12_FDG positive peak in treated HNSCC cells. Cells both positive for 7AAD and C_12_FDG were analyzed within the necrotic and late apoptotic cells, as we suggest they might have been in a senescent state before dying. Cells in G2/M phase of the cell cycle were identified by Hoechst 33342 (Supplementary Figure S2).

### 2.5. Senescence by β-Galactosidase and p21/α Tubulin Staining

The cells were treated identically to the flow cytometry measurement, except for the p21 staining, where the cells were grown on coverslips. For β-galactosidase staining the cells were stained according to the manufacturer’s protocol (Sigma-Aldrich, Taufkirchen, Germany). In short, the washed cells were fixed, washed again, and the cells were incubated overnight in the staining solution at 37 °C. Images were acquired by an inverse Leica DMILLED (Leica, Wetzlar, Germany) microscope. To assess the expression of the p21 and α tubulin proteins an immune staining was performed. The cells were washed and the primary rabbit monoclonal antibodies p21 (Cell signaling Danvers, MA, USA, #2947, 1:200) and mouse α tubulin (Abcam, Cambridge, UK, #ab7291, 1:1000) were incubated overnight at 4 °C. Coverslips were washed and secondary green fluorescence anti-rabbit antibodies Alexa488 and red anti-mouse antibodies Alexa594 (Thermo Fisher Scientific, Waltham, MA, USA) were incubated at 37 °C for 2 h. The cells were counterstained with 4′,6-diamidino-2-phenylindole (DAPI) and mounted in Vectashield (Vector Laboratories, Peterborough, UK). The images were acquired with the Zeiss Axio Imager Z2 fluorescence microscope at 400× magnification (Zeiss, Oberkochen, Germany). Overlays were created using image processing software (Biomas Version 4.1 07/2018, MSAB, Germany).

### 2.6. Clonogenic Survival by Colony Formation Assay

Cells were cultured in T25 flasks for 10 days. Cells were then harvested, and 500 cells were taken from every flask and seeded in 60 mm dishes. Simultaneously, 500 cells were taken from a regularly split culture and seeded in 60 mm dishes. Cell culture medium was replaced after 7 days. After 14 days, cells were fixed and stained with trypan blue. Colonies consisting of at least 50 cells were counted.

### 2.7. Statistics

Graphs were generated using scientific software GraphPad Prism (GraphPad Software, San Diego, CA, USA). Statistical comparison was performed using GraphPad Prism and SPSS (IBM, Armonk, NY, USA). The data of clonogenic assays was analyzed by the unpaired, one-tailed Mann–Whitney U-test. Statistical analysis of the data of flow cytometry was performed by the two-way analysis of variance (two-way ANOVA) to assess the effect of IR, DDRi, and interaction effects. In the text, the term “main effect” refers to the direct effect of the independent variables single IR or single DDRi upon the dependent variables senescence, cell death, and G2 arrest, averaged across the levels of the respective other independent variable. The term “interaction effect” is used for description of combined effect of IR and DDRi on the dependent variables. Additionally to the two-way ANOVA, Holm–Sidak post-hoc multiple comparison test was applied. Homogeneity of error variances was assessed by Brown–Forsythe test. In single cases, where homogeneity of error variances was violated, we indicate it in the text. In these cases, we report only the results of the multiple comparisons and do not interpret the results of the ANOVA. *p*-values for interaction effects detected by ANOVA are stated in the text. In the graphs, *p*-values of Holm–Sidak test are plotted to disaggregate ANOVA results. 

All presented data within this paper are the mean of at least three independent experiments and shown as mean and standard deviation of the mean, unless indicated otherwise. *p*-values < 0.05 were considered statistically significant.

## 3. Results

**Figure 1:**
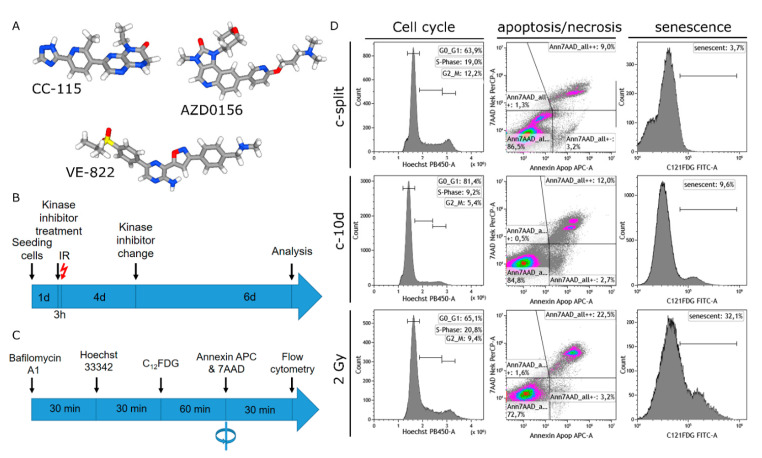
Chemical structures of the used kinase inhibitors, treatment schemes, and gating strategy. Chemical structure of the (**A**) DNA-dependent protein kinase (DNA-PK) and mammalian target of rapamycin (mTOR) inhibitor CC-115, Ataxia telangiectasia mutated kinase (ATM) inhibitor AZD0156 and ataxia telangiectasia and Rad3 related kinase (ATR) inhibitor VE-822. (**B**) Treatment timeline of cell cultures over a period of 11 days. (**C**) The sequence of staining for DNA-content, senescence, apoptosis, and necrosis. (**D**) Gating strategy for cell cycle analysis, apoptosis, and necrosis and senescence in regularly split cultures (c-split), cells seeded for 11 days (c-10d), and cells growing for 10 days after irradiation by 2 Gy.

Two HPV- and two HPV+ HNSCC cell lines and two fibroblast cultures were studied for senescence induction by the DNA-PK and mTOR inhibitor CC-115, ATM inhibitor AZD0156, and ATR inhibitor VE-822 in combination with ionizing radiation (Figure 1A). Cells were treated with a kinase inhibitor and after 3 h cells were irradiated by 2 Gy. Cells could then grow for 10 days (Figure 1B). Cells were stained with BAF 1A and C_12_FDG for senescence detection, with Hoechst 33342 for cell cycle analysis and with Annexin APC and 7AAD for apoptosis and necrosis detection (Figure 1C).

### 3.1. Ability to Perform Homologous Recombination

**Figure 2:**
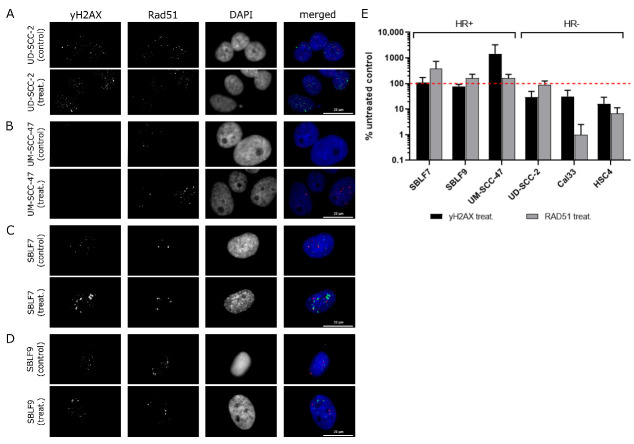
Efficiency of homologous recombination (HR) in two fibroblasts cell cultures, two human papilloma virus (HPV)+ and two HPV- head and neck squamous cell carcinoma (HNSCC) cell lines. Cells were treated with DNA-PK inhibitor CC-115 to inhibit non-homologous end joining (NHEJ) and then irradiated by 10 Gy. (**A**) First column: ionizing radiation (IR)-induced γH2AX foci in UD-SCC-2. Second column: IR-induced Rad51 foci in UD-SCC-2. (**B**) First column: IR-induced γH2AX foci UM-SCC-47. Second column: IR-induced Rad51 foci in UM-SCC-47. (**C**) First column: ionizing radiation (IR)-induced γH2AX foci in SBLF7. Second column: IR-induced Rad51 foci in SBLF7. (**D**) First column: ionizing radiation (IR)-induced γH2AX foci in SBLF9. Second column: IR-induced Rad51 foci in SBLF9. (**E**) Analysis of γH2AX and Rad51 foci after IR in fibroblast cell cultures SBLF7 and SBLF9, HPV+ HNSCC UM-SCC-47 and UD-SCC-2, and HPV- HNSCC HSC4 and Cal33. Compared to the untreated control, Rad51 foci increased upon IR in SBLF7, SBLF9, and UM-SCC-47. SBLF7, SBLF9, and UM-SCC-47 are considered as HR-proficient. Rad51 foci decreased in UD-SCC-2, HSC4, and Cal33. UD-SCC-2, HSC4, and Cal33 are therefore HR-deficient.

The ability of the HNSCC cell lines and fibroblast cell cultures to perform homologous recombination (HR) was studied by an immunostaining-based assay. γH2AX and Rad51 foci were evaluated after cells were treated with CC-115 DNA-PK inhibitor and IR. This treatment aimed to induce DNA DSBs while forcing HR-proficient cells to use HR for their repair. γH2AX marks the appearance of DSBs (Figure 2A). Rad51 functions as a central player in all phases of HR, thus, the count of Rad51 foci should be increased when cells are performing HR (Figure 2B). In HR-deficient cells Rad51 foci should be stable or decline. In comparison to the untreated control, Rad51 foci decreased in UD-SCC-2, HSC4, and Cal33 HNSCC cell lines, indicating them to be HR-deficient (Supplementary Figure S3). Rad51 foci increased in SBLF7, SBLF9, and UM-SCC-47. Healthy human fibroblasts SBLF7 and SBLF9 as well as HPV+ UM-SCC-47 HNSCC cell lines are rated as HR-proficient (Figure 2C).

### 3.2. Induction of Senescence by IR, DDRi, and Combined Treatment

**Figure 3:**
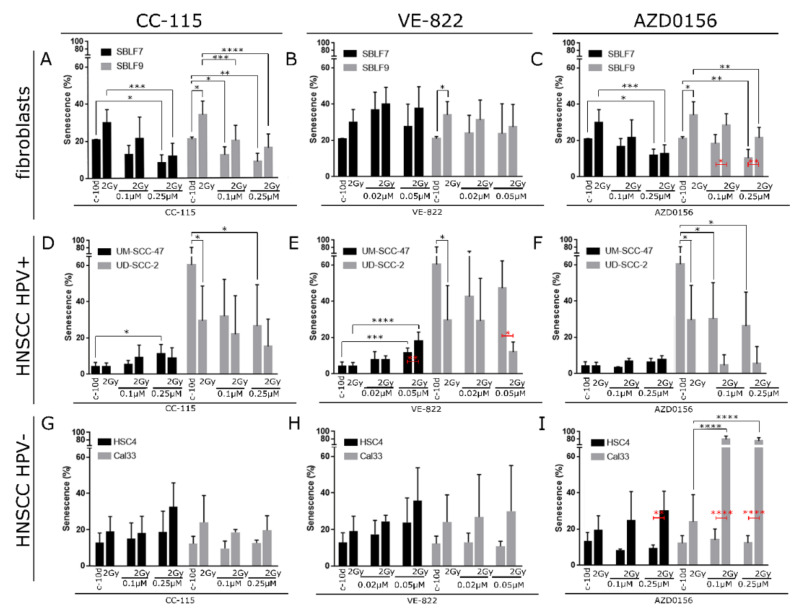
Senescence induction in two fibroblasts cell cultures, two HPV+ and two HPV- HNSCC cell lines. Cells were treated by 2 Gy IR alone, DNA damage response inhibitors (DDRi) alone, or combined IR and DDRi, respectively. (**A**) Fibroblasts cell cultures SBLF7 and SBLF9 treated with DNA-PK and mTor inhibitor CC-115, (**B**) ATR inhibitor VE-822, and (**C**) ATM inhibitor AZD0156. UM-SCC-47 and UD-SCC-2 HPV+ HNSCC cells treated with (**D**) DNA-PK and mTor inhibitor CC-115, (**E**) ATR inhibitor VE-822, and (**F**) ATM inhibitor AZD0156. HSC4 and Cal33 HPV-HNSCC cells treated with (**G**) DNA-PK and mTor inhibitor CC-115, (**H**) ATR inhibitor VE-822, and (**I**) ATM inhibitor AZD0156. Error bars indicate standard deviation. Each experiment was repeated three times at least. *p*-values shown for Holm–Sidak multiple comparisons test in black and red colors. * *p* < 0.05; ** *p* < 0.01; *** *p* < 0.001; **** *p* < 0.0001.

IR alone induced senescence in healthy fibroblasts cell line SBLF9, but not SBLF7. Treatment with CC-115 and AZD0156 decreased senescence levels compared to the control as well as radiation-induced senescence in both fibroblast cell lines. There was no such effect for VE-822 (Figure 3A–C). As homogeneity of error variances was violated for senescence levels in the fibroblast cell lines, we only report the multiple comparison results. 

In HPV+ UM-SCC-47, there was no main effect of IR on senescence and only small effects for all DDRi. VE-822 as main effect increased senescence in UM-SCC-47. For the higher concentration of 0.05 µmol/L, there was an interaction effect of IR and VE-822 which increased senescence for combined treatment (*p* = 0.013). In HPV+ UD-SCC-2, there was a main effect of IR and DDRi CC-115 and AZD0156. In contrast to UD-SCC-2, both IR and DDRi decreased senescence in UD-SCC-2 (Figure 3D–F). Neither the DDRi nor IR were effective in HPV- HSC4 cell line (Figure 3 G–I). In HPV- Cal33, there was an important interaction effect for combined IR and AZD0156 (*p* < 0.0001), leading to a pronounced increase of senescence induction (Figure 3I).

### 3.3. Apoptotic and Necrotic Cell Death and Senescence

We found early apoptotic cells to have a minor impact after the time span of 11 days among all cell lines. Describing cell death, we therefore focus only on necrosis. 

**Figure 4:**
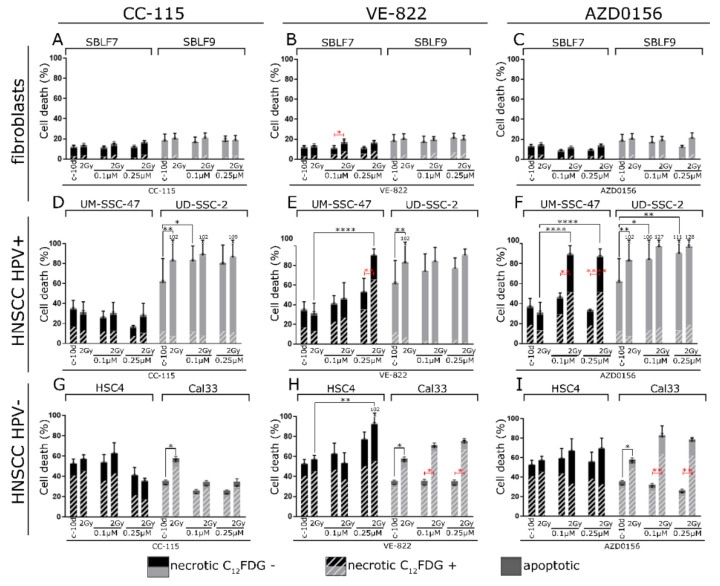
Cell death in two fibroblasts cell cultures, two HPV+ and two HPV- HNSCC cell lines. Distribution of early apoptotic cells, C_12_FDG positive and C_12_FDG negative cells among the necrotic cells. Cells were treated by 2 Gy IR alone, kinase inhibitor alone, or combined kinase inhibitor and 2 Gy IR. Fibroblasts cell cultures SBLF7 and SBLF9 treated with (**A**) DNA-PK and mTor inhibitor CC-115, (**B**) ATR inhibitor VE-822, and (**C**) ATM inhibitor AZD0156. UM-SCC-47 and UD-SCC-2 HPV+ HNSCC cells treated with (**D**) DNA-PK and mTor inhibitor CC-115, (**E**) ATR inhibitor VE-822, and (**F**) ATM inhibitor AZD0156. HSC4 and Cal33 HPV- HNSCC cells treated with (**G**) DNA-PK and mTor inhibitor CC-115, (**H**) ATR inhibitor VE-822, and (**I**) ATM inhibitor AZD0156. Error bars indicate standard deviation for apoptotic cells (grey) and C_12_FDG negative necrotic cells (black). Each experiment was repeated three times at least and controls at least five times. *p*-values shown for Holm–Sidak multiple comparisons test in black and red colors. *p*-values refer to the whole proportion of necrotic cells. * *p* < 0.05; ** *p* < 0.01; *** *p* < 0.001; **** p < 0.0001.

In the fibroblast cell cultures SBLF7 and SBLF9, there were no main effects on necrosis for any of the inhibitors nor IR (Figure 4A–C). 

For HPV+ UM-SCC-47, there were interaction effects of IR and DDRi for AZD0156 (*p* = 0.002) and 0.05 µmol/L of VE-822 (*p* = 0.003). Necrosis was strongly increased upon combined treatment. For HPV+ UD-SCC-2, the assumption of homogeneity of variances was not met. We therefore report Holm–Sidak post hoc test results only. Both single IR and single CC-115 and AZD0156 increased necrosis in UD-SCC-2, but combined IR and DDRi did not lead to increased effect (Figure 4D–F). 

For HPV- HSC4, the assumption of homogeneity of variances was not met. Necrosis was only increased upon VE-822 treatment in Holm–Sidak multiple comparisons test. For HPV- Cal33, there was a main effect of IR only. Although necrosis tended to increase in Cal33 for combined DDRi and IR treatment for AZD0156, no interaction effect could be detected by the two-way ANOVA (*p* = 0.059) (Figure 4G–I).

### 3.4. G2 Arrest

**Figure 5:**
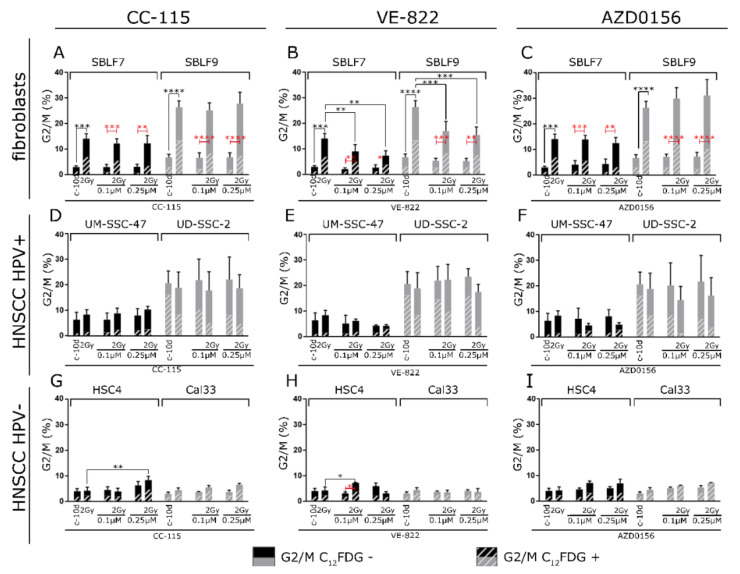
G2/M phase in two fibroblasts cell cultures, two HPV+ and two HPV- HNSCC cell lines. Distribution of C_12_FDG positive and C_12_FDG negative cells among the G2/M cells. Cells were treated by 2 Gy IR alone, kinase inhibitor alone, or combined kinase inhibitor and 2Gy IR. Fibroblasts cell cultures SBLF7 and SBLF9 treated with (**A**) DNA-PK and mTor inhibitor CC-115, (**B**) ATR inhibitor VE-822, and (**C**) ATM inhibitor AZD0156. UM-SCC-47 and UD-SCC-2 HPV+ HNSCC cells treated with (**D**) DNA-PK and mTor inhibitor CC-115, (**E**) ATR inhibitor VE-822, and (**F**) ATM inhibitor AZD0156. HSC4 and Cal33 HPV- HNSCC cells treated with (**G**) DNA-PK and mTor inhibitor CC-115, (**H**) ATR inhibitor VE-822, and (**I**) ATM inhibitor AZD0156. Error bars indicate standard deviation for C_12_FDG negative G2/M cells. Each experiment was repeated three times at least and controls at least five times. *p*-values shown for Holm–Sidak multiple comparisons test in black and red colors. *p*-values refer to the whole proportion of G2/M cells. * *p* < 0.05; ** *p* < 0.01; *** *p* < 0.001; **** *p* < 0.0001.

In both SBLF7 and SBLF9 healthy fibroblasts, IR was very effective as main effect (*p* < 0.0001). Interestingly, a very pronounced G2 arrest was found after 2 Gy IR, persisting even after the 11 days of the assay. DDRi alone did not affect G2 arrest. There was an interaction effect for IR and VE-822 in both SBLF7 (*p* = 0.0108) and SBLF9 (*p* = 0.0018). In the fibroblast cell lines, radiation-induced G2 arrest was abrogated when IR was combined with VE-822 (Figure 5A–C). 

For HPV+ UM-SCC-47 and UD-SCC-2, there was no effect of IR on G2 arrest detectable 11 days after the irradiation. Two-way ANOVA detected a main effect of VE-822 and an interaction effect for AZD0156 and IR for UM-SCC-47 where G2 arrest is reduced upon combined treatment similarly to the fibroblast cell lines (*p* = 0.0218). Nevertheless, here the homogeneity of variances was violated and these results should be interpreted with caution. In general, the G2 arrest in HPV+ HNSCC was more pronounced in the control compared to the HPV- HNSCC (Figure 5D–F).

In HPV- HSC4, there was a main effect for 0.25 µmol/L of CC-115 as well as an interaction effect for 0.02 µmol/L of VE-822 and IR (*p* = 0.0168), both leading to increased G2 arrest. Nevertheless, the effects of the DDRi in the HPV- HNSCC were generally small (Figure 5G–I). 

### 3.5. Background Levels of Senescence and Necrosis 

In order to obtain a better understanding of senescence induction by the 10-day cultivation of cells without splitting, colony formation studies were performed. Regularly split cells were compared with cells cultured for 10 days without splitting before seeding for the colony formation assay. Additionally, senescence and cell death in regularly split cultures and cells cultured for 10 days were assessed by flow cytometry. 

**Figure 6:**
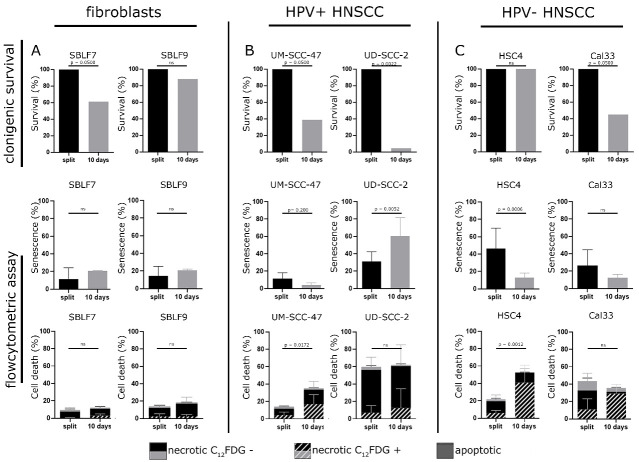
(**A**) First column: fibroblasts, (**B**) second column: HPV+ cell lines, (**C**) HPV- cell lines. First row: clonogenic survival of fibroblast cell cultures SBLF7, SBLF9, HPV+ HNSCC UM-SCC-47, UD-SCC-2, HPV- HNSCC HSC4, and Cal33. Comparison of regularly passaged cells (split) and cells grown for 11 days on a slide (10 days) before seeding for clonogenic assay. Second row: senescence induction assessed by flow cytometry in regularly passaged cells (split) and cells grown for 11 days on a slide (10 days). Third row: apoptosis and necrosis induction assessed by flow cytometry in regularly passaged cells (split) and cells grown for 11 days on a slide (10 days). Distribution of C_12_FDG positive and C_12_FDG negative cells among the necrotic cells. *p*-values refer to the whole proportion of necrotic cells. Error bars show standard deviation. Each experiment was carried out at least three times.

SBLF9 fibroblasts tolerated the 10-day cultivation better than SBLF7 fibroblasts. In flow cytometry, senescence and necrosis did not increase distinctly in the fibroblast cell lines after 10 days of cultivation (Figure 6A). For both HPV+ HNSCC UM-SCC-47 and UD-SCC-2, clonogenic survival decreased for the cells cultured for 10 days. This effect was most pronounced in the UD-SCC-2 cell line. Flow cytometry results propose that this effect in UM-SCC-47 is due to increased necrosis, but due to an important increase of senescence in the UD-SCC-2 cell line (Figure 6B).

Clonogenic survival of HPV- HSC4 was not affected by culturing for 10 days without splitting. Flow cytometry results suggest that decrease of senescence and increase of necrosis outweigh each other. For both HSC4 and Cal33, the background of senescence was decreased after 10 days of cultivation. At the same time, the proportion of necrotic C_12_FDG-positive cells was increased after 10 days (Figure 6C).

### 3.6. Senescence Validation by β-Galactosidase

For histochemical validation of the results observed by flow cytometry, cells were treated as described for the combined C_12_FDG assay and stained by chromogenic SA-β-Gal substrate X-gal after 10 days of cultivation (Supplementary Figure S4). Blue stain should correlate with β-Gal activity and senescence. Blue color was more pronounced for the cell lines and medication combinations where increased β-Gal activity was found by the C_12_FDG assay in flow cytometry. Notably, high levels of senescence cells were detected in flow cytometry for the untreated control in UD-SCC-2 cell line. This was not as clear in X-gal staining for UD-SCC-2 control cells. 

### 3.7. Senescence Validation by p21 and α Tubulin 

p21 levels and alpha-tubulin were assessed by fluorescence microscopy as additional senescence markers (Supplementary Figure S5). For SBLF7, p21 was increased for IR by 2 Gy, VE-822 alone, and combined VE-822 and 2 Gy treatment. For SBLF9, p21 was increased upon 2 Gy alone and combined DDRi and IR, but not for DDRi alone. For UD-SCC-2, p21 decreased when cells were irradiated. The number of remaining UD-SCC-2 cells decreased strongly after IR, which hampered imaging of suitable numbers of cells. In UM-SCC-47, p21 levels were generally low but increased upon VE-822 combined with IR. HSC4 cells expressed p21 but levels did not vary much upon treatment. In the Cal33 cell line, p21 was detectable upon IR as well as DDRi treatment. For AZD0156 and VE-822, cell numbers decreased strongly upon combinatorial treatment with IR, which impeded taking representative images.

## 4. Discussion

We were especially interested in senescence induction by three different DDRi and their combination with IR. A combination of live dyes for C_12_FDG, Hoechst33342, Annexin APC, and 7AAD allowed us to compare the different effects of the inhibitors and IR on senescence, cell cycle control, apoptosis, and necrosis.

The six cell lines reacted very differently upon DDRi treatment. In UM-SCC-47, HSC4, and Cal33, DDRi tended to increase senescence. Interestingly and in contrast to our expectations, senescence induction decreased upon DDRi treatment in the healthy tissue fibroblasts and UD-SCC-2 cancer cell line. Necrosis differed strongly between the cell lines. We found interaction effects for IR and DDRi in single cell lines. In these cases, the combined IR and DDRi enhanced senescence and necrosis induction importantly.

### 4.1. ATR Inhibitor VE-822 and ATM Inhibitor AZD0156 were More Effectively Inducing Senescence and Necrosis for Combinatorial Treatment with IR

The main objective of the present study was to investigate whether combined IR and DDRi will induce senescence more effectively compared to single treatment, among other possible interaction effects. Regarding the induction of senescence, we found interaction for IR and the two DDRi VE-822 (inhibitor of ATR) and AZD0156 (inhibitor of ATM). Their combination with IR led to a pronounced increase of senescence in HPV+ UM-SCC-47 as well as HPV- Cal33. The induction of senescence was especially effective in Cal33, where it led to the inactivation of more than 80% of the living cells. This is particularly promising, as the prognosis of HPV- HNSCC is regarded to be less favorable as the prognosis of HPV+ HNSCC [14]. Besides senescence induction, interaction of VE-822 and AZD0156 with IR also increased necrosis in UM-SCC-47 and Cal33. 

In contrast, IR-induced senescence was decreased upon combined treatment with AZD0156 in healthy human fibroblasts. Additionally, combined treatment did not increase necrosis in the healthy tissue fibroblasts. Combination of IR with VE-822 or AZD0156 therefore seems a promising strategy for cancer treatment. Nevertheless, these effects were observed for single cell lines and no interaction effect could be found for the other two HNSCC cell lines. As the cells were analyzed 10 days after IR in order to focus on senescence induction, we are not able to make a statement on the efficacy of combined treatment concerning apoptosis and early necrosis. A remarkable proportion of Cal33 cells was inactivated by senescence induction upon combinatorial treatment with AZD0156 and IR. Previously, HNSCC with high-risk p53 mutations have been described to assume a senescence-like phenotype and to express increased β-galactosidase staining upon combined cisplatin and Wee-1 inhibitor treatment. Wee-1 inhibition caused override of G2 arrest and sensitized p53 mutated HPV- HNSCC to cisplatin. This was resulting in mitotic arrest and senescence, but not apoptosis [15]. 

A useful additional senescence marker for p53-deficient cancer cells might be the p21. p21 inhibits cyclin-dependent kinases and is involved in the establishment of cell cycle arrest in senescence [16]. An important function of the p21 is to regulate the DDR in senescent cells [17]. Transcriptional activation of p21 is considered one of the most relevant effects of activated p53 for senescence induction [16]. However, it was found that p53-independent activation of p21 is also related to senescence in p53-deficient cells [18]. As p21 can be activated by Chk2 in p53-deficient cells [18], p21 levels might be compromised upon the use of DDRi. In the present study, we found that both HPV+ HNSCC cell lines and p53-mutated HPV- HSC4 and Cal33 express p21 protein. This might indicate their ability to enter senescence despite their lack of functional p53. For UM-SCC-47 and Cal33, the number of viable cells decreased strongly upon VE-822 and AZD0156 in combination with IR, which impeded representative imaging of their p21 protein levels. Together with the high levels for 7AAD in flow cytometry for combination of the ATM and ATR inhibitors with IR, this emphasizes the importance of cell death, among senescence, upon treatment in these cell lines. We therefore suggest that among cell cycle control, both cell death and senescence should be taken into account when studying efficacy of treatment in cancer cells.

### 4.2. DNA-PK/mTor Inhibitor CC-115 did not Interact with IR for Senescence and Necrosis Induction

In contrast to the ATM and ATR inhibitors, we did not find an interaction effect for senescence and necrosis induction for CC-115. CC-115 is a dual mTor/DNA-PK inhibitor which has been described to effectively inhibit NHEJ via its DNA-PK inhibiting properties [19]. DNA-PK inhibitors in combination with IR have previously been tested on HNSCC and have been found to radiosensitize them [20,21,22]. As most studies used clonogenic survival assays, it is not clear whether the tumor cells in these studies have been inactivated by senescence or died by apoptosis or necrosis. Nonetheless, for HPV- Cal33 it has been described that CC-115 strongly induced apoptosis [19]. In the present study, we did not find CC-115 to radiosensitize the HNSCC importantly. Since we set the focus on senescence and therefore analyzed the effects of the inhibitors 10 days after IR, we could not assess the effects on apoptosis and early necrosis. These may be the main effects of the DNA-PK and mTor inhibitor, as it was not an important inductor of senescence. 

Besides the effect on DNA-PK, the inhibition of mTor by CC-115 must also be considered. mTor signaling is involved in the regulation of numerous basic physiological processes such as metabolism, cell survival, and autophagy and is aberrantly overactivated in many cancers [23]. mTor has been reported to be activated in 80–90% of the HNSCC, though mutations in mTor and related upstream genes are particularly prevalent in HPV+ HNSCC [23].

Particularly important for the interpretation of senescence induction is the possible inhibitory effect of mTor on senescence via autophagy. Senescence and autophagy have been reported to be significantly associated in various cell systems [24]. As different studies have come to conflicting results whether autophagy inhibition or activation promotes or delays senescence induction, their interplay is complex and controversial and remains to be further elucidated [25]. Regarding the role of mTor, a number of studies support the idea that mTor signaling promotes senescence [26,27]. Additionally, mTor inhibition has been proposed to increase lifespan and to reduce age-related diseases [28]. The mTor-inhibiting component of CC-115 might therefore counteract senescence induction by DNA-PK inhibition. This might be an explanation for the low levels of senescence we observed upon CC-115 treatment.

### 4.3. Induction of G2 Arrest in the Six Cell Lines

Regarding cell cycle distribution, IR clearly induced a G2 arrest in the healthy fibroblast cell cultures, which persisted even after 10 days. Neither IR nor DDRi induced necrosis in the fibroblast cell lines. IR induced senescence in the SBLF9 fibroblast cell line. This may indicate that these normal tissue cells can manage DNA damage and combined treatment relatively well. We expect them to have intact cell cycle checkpoints, carrying out repair processes and inactivating unrepairable cells in a controlled manner by entering senescence. In contrast to this, in the four cancer cell lines the induction of necrosis and senescence varied largely, while there was nearly no additional G2 arrest induced by 2 Gy IR or combined treatment. This may indicate that the DNA damage processing is impaired among these cancer cells.

Particularly HPV+ HNSCC have been characterized as highly radiosensitive due to inefficient DSB repair and prolonged arrest in G2 [5]. We expected HPV+ cells to show more pronounced G2 arrest upon IR, but could not detect enhanced G2 arrest in any of the HNSCC 10 days after 2 Gy IR. A possible explanation is that the effect of IR on G2 arrest was analyzed more shortly after IR in other studies and might have worn off within the 10 days after IR. For comparison, for UD-SCC-2, the proportion of G2 arrest upon 6 Gy IR has been described to be major 24 h after IR and to have started to decrease already after 48 h [29].

### 4.4. Radiosensitization in the Context of Abrogation of G2 Arrest 

In the present study, we found 0.05 µmol/L of VE-822 to distinctly abolish radiation-induced G2 arrest in healthy fibroblasts SBLF7 and SBLF9. There was a clear interaction effect of IR and the ATR-inhibitor on G2 arrest in these cell lines. For UM-SCC-47, G2 arrest tended to decrease similarly as in the fibroblast cell lines for VE-822 as well as AZD0156. Two-way ANOVA detected an interaction effect on G2 arrest for AZD0156 and IR for UM-SCC-4, and a main effect of single VE-822. Importantly, it has to be stated that the assumption of homogeneity of error variances was not met for G2 arrest in UM-SCC-47, so these results have to be treated with caution. We found this diminishing effect of combinatorial treatment on G2 arrest exclusively in the fibroblast cell lines and UM-SCC-47. These are the three cell lines we found to be HR-proficient. 

Several studies support the idea that sensitization achieved by inhibition of ATR/Chk1 is related to impairment of HR [30]. Accordingly, radiosensitization by ATR-inhibitor VE-822 has been found to correlate with persistent γH2AX foci but decreased Rad51 expression [31]. It was also reviewed that radiosensitization provoked by ATR/Chk1 inhibition can be related to abrogation of the G2 checkpoint, especially for p53-deficient cells [30]. VE-822 has been found to increase G1 arrest and to abolish radiation-induced G2 arrest in pancreatic cancer cells [31]. Specific inhibition of Chk1 by several inhibitors has been shown to abrogate radiation-induced G2 arrest and to cause radiosensitization in HNSCC [29,32,33]. HPV+ HNSCC have been found to be more affected by this than HPV- HNSCC [29].

In our study, abrogation of G2 arrest after ATR inhibition in HR-proficient fibroblast cell lines did not lead to an increase in their sensitivity to IR, as there was no corresponding induction of senescence or necrosis. We found induction of senescence and necrosis as a result of radiosensitization in HR-proficient UM-SCC-47, but although our data suggest this might be related to abrogation of G2 arrest, this is ambiguous due to violation of homogeneity of variances. Regarding these results and the fact that ATM and ATR have been related to both HR and NHEJ [2,34], we cannot reaffirm the connection between impairment of G2 arrest and HR. 

### 4.5. HR Status Did Not Have Major Influence on Senescence Induction upon IR and DDRi Treatment 

We sought to assess clear differences upon DDRi and IR treatment between HR-proficient and HR-deficient cells. In contrast to our expectations, we did not find exclusive or major efficacy of ATR inhibition in HR-proficient cell lines, nor strictly HR-dependent sensitization to IR by abrogated G2 arrest. We can only state that the studied DDRi did not induce cell death or senescence in HR-proficient healthy fibroblasts, and that senescence levels in HR-proficient UM-SCC-47 were generally low and increased only slightly upon treatment. A reason for the absence of clear differences between HR-proficient and HR-deficient cell lines could lie in the complexity of the DDR pathways. ATM and ATR have been related to both HR and NHEJ [2,34], have overlapping effects, and share many substrates [30]. DNA-PK is related to NHEJ exclusively [2], however the used inhibitor is a DNA-PK and mTor inhibitor.

### 4.6. Individual Reactions and Sensitivity towards DDRi Treatment in HPV+ and HPV-Cell Lines 

Besides the influence of HR-proficiency, we sought to identify differences in the reaction towards DDRi among HPV- and HPV+ HNSCC. Nevertheless, we found that the effects of irradiation and inhibitor treatment on senescence and necrosis varied rather individually for each cell line than in a HPV-dependent manner.

HNSCC UM-SCC-47 and UD-SCC-2 are both HPV-positive. Nevertheless, upon treatment with DDRi CC-115 and VE-822, they acted reversely. The DDRi led to senescence induction in UM-SCC-47, but decreased senescence levels strongly in UD-SCC-2. Regardless of their HPV status, each HNSCC cell line was sensitive to different DDRi. Out of the two cell lines we found to react with important increase of senescence or necrosis upon combinatorial DDRi and IR treatment, one was HPV+, the other HPV-. Our findings suggest that HNSCC cell lines act rather individually upon treatment with IR and combined inhibitor treatment. A possible explanation is that there might be more relevant modifications existing in the cells, which should be taken into account besides the HPV status.

### 4.7. Molecular Heterogeneity as the Cause for Varying Efficacy of DDRi in HNSCC Cell Lines

The TP53 has been found to be one of the most frequently mutated genes in HNSCC [35], with high-risk or low-risk TP53 mutations causing variant sensitivity to IR and chemotherapeutic drugs [36]. HPV- HNSCC Cal33 and HSC4 are p53 mutated [10]. As tumor suppressors p53 and pRB are disabled by viral oncoproteins in HPV+ HNSCC, these cells are functionally p53-deficient [5,29].

Compromising p53 and pRb by viral oncoproteins might be a feature allowing HPV+ HNSCC to avoid senescence in order to drive the process of carcinogenesis [37]. Similarly, for HPV-HNSCC, mechanisms counteracting senescence have been described [37,38,39]. Nevertheless, various studies have shown that cancer cells enter senescence in spite of mutations in the TP53, p16 or p53 deficiency or proof of papillomavirus oncoproteins. It was therefore proposed that although being positive regulators for senescence, p53, p21, and p16 are not essential for senescence [40]. We therefore propose that the individual functionality of p53, p16, and pRb in each HNSCC cell line might influence their propensity to enter senescence and could be a main factor underlying the diverging results we observed for senescence induction. As the mutational status of the TP53 affects the functionality of the different DDRi pathways, this might also influence the general efficacy of DDRi inhibitors. It has been found that efficacy of ATR inhibition depends on the functionality of ATM-p53 signaling: upon combined cisplatin and VE-821 ATR-inhibitor treatment, DSB accumulate in a dysfunctional S phase, which later is made up for by a compensatory S phase checkpoint activated by ATM [41]. Similarly, efficacy of CC-115 has been found to be major in ATM-deficient cells [19]. In HNSCC, mutations in ATR (4–10%) and ATM (1–16%) are common [36]. Among the mutational status of the TP53, other mutations concerning DDR pathways might therefore influence the individual efficacy of DDRi in HNSCC. 

### 4.8. Decreased Senescence Levels upon DDRi Treatment can be Explained by Involvement of DDRi in Senescence Induction

We expected combinatorial IR and DDRi treatment to induce senescence. IR evokes DSB [2], which then cannot be repaired due to the inhibition of DDR pathways. Particularly irreparable DSB have been found to induce senescence previously [42,43,44]. In clear contrast to these expectations, we found senescence to be generally decreased in the UD-SCC-2 cell line upon DDRi treatment. Similarly, senescence was decreased in the healthy fibroblast cell cultures for treatment with DDRi AZD0156 and CC-115. Nonetheless, in HNSCC cell lines UM-SCC-47 and Cal33, senescence was induced for the same DDRi, as we would have expected for all tumor cell lines. For a better understanding of these contradictory effects of DDRi on senescence induction, one must consider the fact that DDR pathways are in many cases directly involved in senescence induction and maintenance [24]. Strategies developed by cancer cells to evade senesce therefore often involve impairment of DDR pathways [37,38,39]. Various therapies aim to restore the capacity to senesce in cancer cells. As an example, overexpression of Chk2 was found to help restore senescence in some p53-deficient cancer cells [18,45]. In line with this finding, inhibition of ATR/ATM or Chk1/Chk2 was found to delay the onset of replicative senescence [24]. These findings may explain the decrease in senescence we observed upon inhibition of ATM in the fibroblast cell cultures and UD-SCC-2.

To conclude, the inhibition of key factors of the DDRi might have different outcomes: on the one hand, DDRi will lead to persisting DNA damage which can lead to senescence induction [42,43,44]. On the other hand, inhibiting some key factors of the DDR might possibly compromise the onset of senescence in some cases. Both the choice between p53, p16/pRb, and other pathways to enter senescence as well as the ability of each pathway to induce senescence are cell-type-specific or even vary within the same type of cell [42]. Whether senescence is induced or disturbed upon DDRi treatment, might therefore partly rely on the preferences of the cell lines for the engagement of one pathway or another for senescence induction. 

## 5. Conclusions

Our main objective was to investigate whether combined IR and DDRi will induce senescence more effectively compared to single treatment. We found interaction effects for IR and DDRi for the ATM and ATR inhibitor in single cell lines. In these cases, the combined IR and DDRi enhanced senescence and necrosis induction importantly. In contrast to the ATM and ATR inhibitor, the DNA-PK and mTor inhibitor was not a major inductor of senescence and did not interact with IR. 

In single cell lines, particularly the induction of senescence led to an inactivation of an important proportion of the cells. Therefore, it is important to study senescence in addition to apoptosis, necrosis and cell cycle control. The fibroblast cell lines generally tolerate IR or combined treatment better than the tumor cell lines. Varying results were found for the HNSCC, probably as a result of molecular heterogeneity, complex interplay of the DDR pathways, and the involvement of DDR pathways in senescence induction. Both HPV status and HR activity appear to have a limited role in the application of DDRi. Since we found complex differences in tolerance of the combined DDRi and ionizing radiation treatment of HNSCC cell lines, further studies are needed to better understand the differences in the processing of DNA damage compared to normal tissue cells. For better comparability healthy mucosal epithelial keratinocytes should be used as controls. 

## Supplementary Materials

The following are available online at https://www.mdpi.com/2073-4409/9/9/2012/s1.
